# A226 THE EFFICACY OF MEDICAL THERAPY TO PREVENT SURGERY AND IMPROVE OUTCOMES AMONG PATIENTS WITH STRICTURING CROHN’S DISEASE: A SYSTEMATIC REVIEW

**DOI:** 10.1093/jcag/gwae059.226

**Published:** 2025-02-10

**Authors:** Q Alkhateeb, A Saad, E Squirell

**Affiliations:** Gastroenterology, Queen’s University, Kingston, ON, Canada; University of Ottawa, Ottawa, ON, Canada; Gastroenterology, Queen’s University, Kingston, ON, Canada

## Abstract

**Background:**

Despite the advancements in inflammatory bowel disease (IBD) management, the percentage of Crohn’s Disease (CD) patients presenting with obstructive symptoms due to small and large bowel strictures is estimated to be 25% and 10%, respectively. Treatment of CD has advanced over the past 20 years to include safer, more targeted options that carry less morbidity than surgical intervention. However, the efficacy of medical therapy once fibrosis has begun is theoretically reduced and, until recently, has had limited study.

**Aims:**

The aim of this systematic review is to examine the efficacy of medical therapies, such as biologics (TNF inhibitors, interleukin inhibitors, anti-integrin therapy) and immunomodulators (thiopurines and methotrexate) to avoid the need for surgical intervention in stricturing Crohn’s disease.

**Methods:**

We searched Medline, Embase, the Cochrane library, and Web of Science for experimental (i.e., randomized and non-randomized controlled trials), quasi-experimental (i.e., interrupted time series) and observational studies (i.e., cross-sectional surveys) examining the effect of medical therapies among adult and pediatric CD patients admitted for the management of strictures. Records were screened, in duplicate and independently, by their title and abstract and then full-text and relevant data were extracted using a standardized data extraction sheet. Efficacy data were pooled using meta-analyses, when applicable, or synthesized narratively.

**Results:**

Our preliminary search yielded a total of 1059 unique records and 22 studies (3 RCTs, 19 cohort studies) were included in our analysis. Results from 8 cohort studies show that among 546 patients receiving TNF antagonists, 80% responded to treatment and did not require surgery at an average follow-up period of 4.1 years (95% CI 71% to 88%). Furthermore, one RCT and one cohort study found that Azathioprine in combination with anti-TNF therapy improved obstructive symptoms and reduced the odds of hospitalization and surgery by half.

**Conclusions:**

Our review highlights systemic medical therapy, namely TNF antagonists, as a promising solution to prevent the need for surgery among patients with stricturing Crohn’s disease. More robust research is needed to confirm these findings and compare their efficacy.

**Funding Agencies:**

None

